# Approach to undifferentiated dyspnea in emergency department: aids in rapid clinical decision-making

**DOI:** 10.1186/s12245-018-0181-z

**Published:** 2018-04-04

**Authors:** Siva Nageswara Rao Guttikonda, Kiran Vadapalli

**Affiliations:** Department of Internal Medicine, Rangaraya Medical College, Government General Hospital, Raja Ram Mohan Rai road, Kakinada, Andhra Pradesh 533001 India

**Keywords:** Dyspnea, Multiorgan USG, Emergency department, Diagnostic strategy, Resource limited setting

## Abstract

**Background:**

Diagnosis and management of patients presenting with acute dyspnea is one of the major challenges for physicians in emergency department (ED). A correct diagnosis is frequently delayed and difficult to ascertain, and clinical uncertainty is common, explaining the need for rapid diagnosis and a management plan. The primary aim of our study is to assess a diagnostic strategy using multiorgan point of care ultrasonography (USG) to differentiate patients presenting with acute dyspnea to ED into different diagnostic categories for timely management in a resource-limited setting.

**Methods:**

This is a prospective cohort study which assessed the diagnostic performance of a strategy in evaluating patients presenting with undifferentiated dyspnea as primary predominant complaint to ED. Focused multiorgan USG which includes cardiac USG for left ventricle systolic function, right ventricle enlargement, and pericardial effusion, inferior vena cava (IVC) diameter and collapsibility, lung USG to identify various patterns (acute interstitial syndrome, pneumothorax, pleural effusion, consolidation, etc.) and renal USG to assess kidney size and echotexture was performed. Later, patients were grouped into one of ten clinical syndromes defined in the study based on USG and clinical patterns. Emergency diagnosis was compared with final hospital diagnosis to assess the accuracy of this strategy.

**Results:**

Concordance between ED diagnosis of dyspnea using the diagnostic strategy proposed in the study with final hospital diagnosis was high with agreement in 88% of patients (Kappa statistic = .805, *p* = .000) which is statistically significant. The most common diagnosis was acute decompensated heart failure (ADHF). Sensitivity and specificity of the diagnostic strategy used in this study to identify ADHF was 97.3 and 93.3%, respectively. On multivariate analysis, jugular venous distension, fever and cough, ejection fraction (by eyeball method), dilated IVC, absent to decreased lung sliding showed independent association in predicting cardiac and non-cardiac diagnosis.

**Conclusions:**

The present study concludes that integrating focused multiorgan USG by lung-cardiac-IVC and renal ultrasound into routine clinical evaluation of patients with dyspnea has a higher accuracy for differentiating causes of dyspnea in emergency department. This strategy can be adopted even in resource limited setting.

## Background

Acute dyspnea is one of the main reasons for admission to the emergency department (ED) [1]. Physicians working in the ED often need to make a rapid diagnosis and devise a treatment plan on the basis of limited clinical information [2, 3]. Rapid and accurate diagnosis and management can be lifesaving for patients with acute dyspnea [4]. However, making a differential diagnosis and selecting early treatment for patients with acute dyspnea in the ED is a clinical challenge that requires complex decision-making in order to achieve hemodynamic balance, improve functional capacity, and decrease mortality and the length of hospital stay [5]. Methods for evaluation of emergency patients with possible acute decompensated heart failure (ADHF) include the history, physical examination, chest radiography, 12-lead electrocardiography (ECG), and measurement of brain natriuretic peptide (BNP) or N-terminal pro-BNP [6]. The physical examination, even with the addition of chest radiography and ECG, is often imprecise and simply starting “dual therapy” for ADHF and chronic obstructive pulmonary disease (COPD) can be harmful [7].

Recently, there has been interest in utilizing chest USG for the diagnosis of acute respiratory failure in ICU. Bedside Lung USG in Emergency (BLUE) protocol proposed by Lichenstein et al. is a landmark study in this regard [8]. There have been few ED studies demonstrating the role of multiorgan point of care USG to evaluate dyspnea. Kajimoto et al. were the first to demonstrate the screening potential of rapid evaluation by lung-cardiac-inferior vena cava (LCI) integrated ultrasound for differentiating ADHF from primary pulmonary disease in patients with acute dyspnea in the emergency setting [9]. Russel et al. used LUCUS protocol to diagnose ADHF in patients with undifferentiated dyspnea in ED [10]. Later, Pirozzi et al. and Gallard et al. evaluated adding cardiopulmonary USG to routine clinical examination to manage dyspnea patients in ED [11, 12]. Most of the above studies focused on assessing the diagnostic performance of USG in identifying ADHF in patients presenting with dyspnea to ED, leaving behind non-cardiac causes of dyspnea, thereby necessitating a strategy to differentiate various non-cardiac causes of dyspnea. No studies were performed in resource-limited ED setting. The primary aim of our study was to assess a diagnostic strategy using multiorgan point of care ultrasonography (USG) to distinguish patients presenting with acute dyspnea to ED into different diagnostic categories for timely management in a resource-limited setting.

## Methods

### Study design and setting

This was a prospective cohort study which assessed the diagnostic performance of a strategy using multiorgan point of care USG in evaluating patients presenting with dyspnea in emergency care. This study was done at emergency department (ED) at Government General Hospital, Kakinada, between June 2016 and December 2016. This study protocol was approved by the Institutional Ethical Committee. A written informed consent was obtained from all patients.

#### Selection of patients

Patients who were 16 years and older, visiting the ED with undifferentiated dyspnea as primary predominant complaint (either sudden onset dyspnea or increase in severity of chronic dyspnea) were included in the study within 1 h of their arrival. Undifferentiated dyspnea was defined for the purpose of this study as two or more possible etiologies in the differential diagnosis according to their treating clinician. Patients with definite etiology of dyspnea which would include a patient with known heart failure not compliant with medication or diet restrictions, or known asthmatics who responded to bronchodilators as per the treating clinician, or patients in whom the treating clinicians were confident in their diagnoses after initial assessment (e.g., patients in whom ECG shows ST elevation MI) were excluded.

### Clinical evaluation

Patient’s medical history, vital signs, and systemic examination were recorded by the enrolling physician. Dyspnea at admission was measured using a 5-point LIKERT scale in sitting position. All patients underwent routine tests as part of the hospital protocol (ECG, chest X-ray, and labs). The clinical probability of heart failure and obstructive lung disease was noted using modified Boston criteria and GOLD clinical criteria of COPD, respectively [13–15].

### Multiorgan point-of-care USG

After initial evaluation, all patients were subjected to a focused multiorgan USG at bedside using a standard medium frequency curved array probe which includes the following.

#### Cardiac USG

Parasternal long- and short-axis views—left ventricular ejection fraction (EF) was estimated visually in the parasternal long-axis view by wall contraction and thickening. EF was confirmed in the parasternal short-axis view at the level of the papillary muscles. Mitral valve E-point septal separation (EPSS) is the distance from the anterior mitral valve leaflet, and the ventricular septum in early diastole measured in *M* mode is also noted for every patient. Left ventricular systolic function was typically graded as normal (EF > 50%), moderate dysfunction (EF 30–50%), or severe dysfunction (EF < 30%) basing on eyeball visual estimate and EPSS measurement. A qualitative evaluation of the right ventricle (RV) dimension was made for RA/RV dilatation, considering RV/LV-end diastolic diameter > 0.9 in the AP4 view as abnormal.

#### Lung USG

Lung USG was performed on each hemithorax divided into five zones (two anterior, two lateral, and one posterior) with patient in seated or lying down position. Several signs were explored to conclude on typical patterns as per the international evidence-based recommendations on point-of-care lung USG [16] which includes lung sliding, pleural effusion (anechoic space between parietal and visceral pleura with sinusoid sign), pneumothorax (loss of lung sliding with positive lung point), and acute interstitial syndrome (AIS), defined as B-pattern with at least three B-lines in two lung zones bilaterally and lung consolidation signs (focal B-lines with tissue-like echotexture and dynamic air bronchograms) [16, 17].

#### IVC USG

IVC USG was performed in sub-xiphoid view for diameter and collapsibility. An IVC with a maximal diameter of ≥ 2 cm and < 50% collapse was considered plethoric. An IVC with a maximal diameter of ≤ 2 cm and > 50% collapse was considered collapsible.

#### Renal USG

Renal USG was performed using an anterior subcostal approach using the liver as a sonographic window for the right kidney and an intercostal approach for the left kidney. Maximal longitudinal axis length and echogenicity of the kidneys were noted.

**Fig 1:**
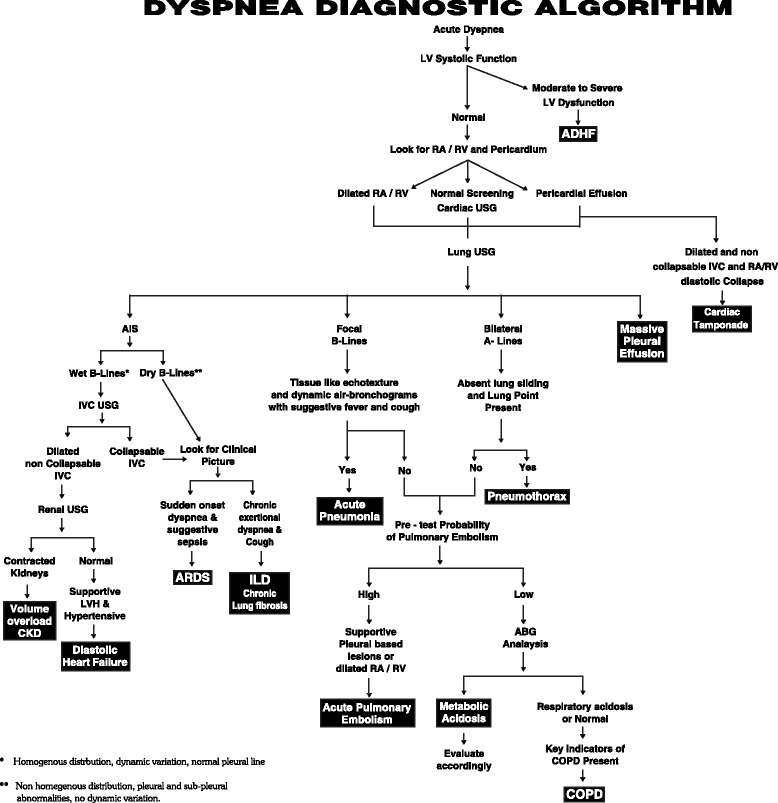
Dyspnea diagnostic algorithm

After a complete assessment, patients were assigned into one of the ten clinical syndromes defined as mentioned in Table 1 by following the constructed algorithm mentioned in Fig. 1. Patient’s time for improvement in their symptom status was noted.

**Table 1:** Clinical syndromes basing on USG patterns and clinical variables

ADHF (systolic dysfunction)	Acute interstitial syndrome (the presence of multiple diffuse bilateral B-lines) with LV dysfunction on cardiac USG
Acute pneumonia	Lung USG showing focal B-lines, subpleural echo poor region with tissue-like echotexture and dynamic air bronchogram with normal LV function.
ARDS	Acute interstitial syndrome (presence of multiple diffuse bilateral B-lines) in non-homogenous distribution and anterior subpleural consolidation, reduced lung sliding normal LV function with suggestive clinical presentation (sudden onset and suggestive sepsis).
COPD or Obstructive airway disease	Bilateral A-lines with decreased lung sliding on lung USG with normal LV function and normal kidneys with key indicators of COPD present clinically.
Acute Pulmonary Embolism	Normal LV function, A-lines on lung USG with or without focal B-lines, dilated RV, dilated IVC with low collapsibility index with high pre-test probability of PE.
Chronic lung disease (ILD or chronic lung fibrosis)	Normal LV function, irregular fragmented pleural line, subpleural abnormalities, bilateral B-lines in non- homogenous distribution with or without dilated RV and dilated IVC with low collapsibility index with suggestive clinical picture (h/o chronic exertional dyspnea and cough).
Diastolic Heart Failure	Normal LV function with LV hypertrophy (LVH), left atrial enlargement, multiple diffuse bilateral B-lines, dilated IVC with low collapsibility index with suggestive clinical presentation (e.g., hypertensive, diabetic)
Volume overload	Normal LV function, normal RV, multiple diffuse bilateral B-lines, dilated IVC, contracted kidneys.
Pneumothorax	Normal LV function, bilateral A-lines with absent lung sliding with observed lung point with suggestive clinical picture.
Tamponade	Normal LV function, pericardial effusion with dilated and non-collapsible IVC and RA/RV diastolic collapse with suggestive clinical signs.

The final hospital diagnosis of patients was determined by two independent physicians, a cardiologist and a pulmonologist, using the reference standard definition for heart failure and pulmonary diseases in accordance with routine standard evaluation using chest X-ray, echocardiographic examination, cardiac functional assessment (exercise test), pulmonary function test, full blood count, biochemistry, and invasive investigation or angiography without knowledge of the USG data collected in ED. The initial ED diagnosis is compared with final hospital diagnosis as mentioned on discharge sheet.

### Statistical analysis

All data were analyzed using the SPSS V.20.0.0 software package. A *P* value < 0.05 is regarded as statistically significant. The comparison of the group of patients who have cardiac and non-cardiac diagnosis was done using Student’s *t* test and chi-square test for continuous and categorical variables, respectively. The performance of different diagnostic tools was analyzed using sensitivity and specificity analysis. The level of agreement between ED diagnosis and final hospital diagnosis was assessed using Cohen’s Kappa test.

## Results and discussion

We enrolled a total of 108 patients in the study. Of these, 8 patients were excluded as they left the hospital against advice. So, a total of 100 patients were included in the data analysis.

### Patient characteristics

The mean (± SD) age among dyspnea patients was 50 (± 15.85) years with a minimum age of 16 years and maximum age of 90 years. Age stratified ratio according to ≥ 60:< 60 years is 36:72 showing elderly patients constituted only 36% of our sample. All the patients in our study were categorized into three groups basing on the diagnosis. The groups were cardiac, non-cardiac (includes pulmonary, renal, and metabolic/systemic causes), and with combined cardiac and non-cardiac cause of dyspnea. Distribution of our patients according to category of diagnosis was cardiac constituting 43%, non-cardiac 29%, and patients with both cardiac and non-cardiac conditions 28%. The mean age of presentation of cardiac patients was 53 (± 16.16) years, and non-cardiac patients was 45 (± 14.05) years (Table 2).

**Table 2:** Characteristics of patients according to category of diagnosis (continuous variables) (*N* = cardiac 43, non-cardiac 29, both 28)

	Cardiac patients mean ± SD and (95% CI)	Non-cardiac patients mean ± SD and (95% CI)	Both mean ± SD and (95% CI)	*P* value
Age	53.55 ± 16.16 (48.58–58.05)	45.31 ± 14.05 (39.36–50.6)	49.53 ± 16.31 (43.2–55.8)	0.093
Systolic blood pressure	139.302 ± 31.04 (129.74–148.85)	119.65 ± 25.28 (110.03–129.27)	143.57 ± 45.88 (125.78–161.63)	0.002
Diastolic blood pressure	85.81 ± 15.77 (80.96–90.68)	74.82 ± 13.26 (69.78–79.87)	91.42 ± 27.31 (80.83–102.02)	0.005
Heart rate	111.465 ± 20.83 (105.03–117.08)	117.00 ± 31.15 (105.14–128.24)	113.89 ± 21.53 (105.54–122.24	0.642
Respiratory rate	28.44 ± 4.22 (27.14–29.74)	33.138 ± 6.610 (30.62–35.65)	30.536 ± 3.2028 (29.29–31.77)	0.00
SpO2	89.93 ± 8.90 (87.18–92.62)	82.964 ± 13.16 (77.85–88.07)	80.82 ± 14.46 (75.21–86.42)	0.004
IVC maximum diameter	18.91 ± 3.87 (17.71–20.10)	10.46 ± 6.89 (7.84–13.08)	17.17 ± 5.147 (15.17–19.16)	0.00
IVC minimum diameter	12.86 ± 4.53 (11.46–14.25)	5.26 ± 5.50 (3.17–7.36)	11.06 ± 5.993 (8.74–13.38)	0.00
LV systolic diameter in PLAX	3.85 ± 1.213 (3.48–4.22)	2.82 ± 0.583 (2.60–3.04)	3.56 ± 0.975 (3.18–3.94)	0.00
EPSS	12.007 ± 6.80 (9.912–14.10)	3.624 ± 1.70 (2.97–4.27)	9.039 ± 5.93 (6.73–11.34)	0.00
Kidney size	9.60 ± 0.667 (9.39–9.80)	9.57 ± 1.36 (9.05–10.05)	8.96 ± 1.16 (8.50–9.41)	0.031
Blood urea nitrogen	39.69 ± 13.06 (35.67–43.71)	57.65 ± 43.71 (41.02–74.28)	65.42 ± 30.97 (53.41–77.43)	0.001
Serum creatinine	1.295 ± 0.66 (1.09–1.51)	3.093 ± 3.58 (1.72–4.45)	3.476 ± 2.669 (2.46–4.53)	0.00
Time to relief from dyspnea in Hrs	22.64 ± 18.85 (16.36–28.93)	18.00 ± 21.015 (4.64–31.35)	32.00 ± 21.91 21.09–42.90)	0.139
Likert scale	2.816 ± 0.729 (2.57–3.05)	2.87 ± 1.027 (2.26–3.45)	3.3 ± 0.656 (2.99–3.60)	0.075
Hospital LOS	6.034 ± 3.109 (5.07–6.99)	4.86 ± 4.434 (3.22–6.51)	7.56 ± 5.998 (5.24–9.89)	0.01
Modified Boston Criteria for HF	8.07 ± 1.334 (7.65–8.48)	6.86 ± 1.156 (6.442–7.302)	7.893 ± 1.065 (7.48–8.306)	0.00

*SD* standard deviation, *CI* confidence interval, *IVC* inferior vena cava, *LV* left ventricle, *EPSS* E-point septal separation, *LOS* length of stay, *HF* heart failure

### Distribution of patients according to diagnosis

Patients with ADHF constituted 43%, and patients having both cardiac and non-cardiac cause of dyspnea constituted 28% mostly with a diagnosis of ischemic cardiomyopathy (ICMP) with chronic kidney disease (CKD), COPD, or interstitial lung disease (ILD) with RV failure, and ADHF with pneumonia. The remaining patients had other causes like ARDS (7%), COPD (4%), acute pulmonary embolism (7%), acute pneumonia (4%), volume overload (4%), and massive pleural effusion (3%) (Table 3).

**Table 3:** Distribution of patients according to diagnostic category

Emergency department diagnosis	Number of patients	Number of incorrect ED diagnosis	Final discharge diagnosis of incorrect ED diagnosis cases
ADHF (systolic or diastolic heart failure)	43	2	1. Volume overload/AKI 2. Acute bilateral bronchopneumonia
COPD	4	Nil	
ARDS	7	1	Anaphylaxis
Acute pulmonary embolism	7	4	1. ASD/Beri beri 2. Pneumonia/sepsis with DIC 3. ADHF/pneumonia/severe anemia 4. ILD with Ac exacerbation of PAH
Acute pneumonia	4	1	Severe anemia with ADHF
Volume overload (AKI, CKD)	4	Nil	
Massive pleural effusion	3	Nil	
Both (cardiac and non-cardiac)	28	3	1. IPF acute exacerbation/RV failure 2. ADHF/malignant effusion 3. Severe anemia/RPGN

*ADHF* acute decompensated heart failure, *AKI* acute kidney injury, *ASD* atrial septal defect, *COPD* chronic obstructive pulmonary disease, *DIC* disseminated intravascular coagulation, *ARDS* acute respiratory distress syndrome, *ILD* interstitial lung disease, *PAH* pulmonary arterial hypertension, *IPF* idiopathic pulmonary fibrosis, RPGN rapidly progressive glomerulonephritis

On univariate analysis of clinical variables recorded at the time of admission, history of orthopnea, fever and cough, past history of CAD, jugular venous distension, and displaced apex beat, modified Boston criteria for HF score showed significant difference between the diagnostic categories. Among the patients with absent to decreased lung sliding, 77% belonged to non-cardiac group and 23% belong to cardiac group which is significant difference between the diagnostic groups (*p* = .002). B-profile did not show significant difference between the groups. It was observed in 72% of cardiac patients while 41% of non-cardiac patients also showed B-profile. EF visual estimate correlated well with final diagnosis with only 2 non-cardiac patients given false positive LV dysfunction (Table 4). IVC diameter between cardiac (mean 18.91 ± 3.87 mm) and non-cardiac groups (mean 10.46 ± 6.89 mm) showed significant difference (Table 2). On multivariate analysis, only jugular venous distension, ejection fraction (by eyeball method), dilated IVC (for cardiac), h/o fever and cough, and absent to decreased lung sliding (for non-cardiac) showed independent association in predicting cardiac and non-cardiac diagnosis.

**Table 4:** Characteristics of study patients according to category of diagnosis (categorical variables) (*N* = cardiac 43, non-cardiac 29. Both cardiac and non-cardiac 28)

Variable	Category of diagnosis			
	Cardiac	Non-cardiac	Both	*P* value
Exertional dyspnea	23	6	13	.019
Orthopnea	29	8	17	.003
Paroxysmal nocturnal dyspnea	9	2	3	.204
Previous heart failure	6	0	2	.093
Chronic kidney disease	0	0	4	NA
Chronic respiratory disease	2	0	3	.185
Coronary artery disease	5	0	0	.031
Fever and cough	2	16	12	.000
Any cardiac murmur	1	0	1	.616
Peripheral edema	23	7	11	.045
Jugular venous distension	18	1	7	.001
Displaced apex beat	10	0	4	.020
S3	3	0	0	.133
Wheeze	3	6	5	.203
Rhonchi and rales	0	2	3	.110
Basal rales	13	3	6	.135
Rales > 1/3 lung fields	4	3	8	.060
ECG abnormality	27	10	20	.011
Lung sliding (absent to decreased)	3	10	0	.002
Pleural effusion	3	3	3	.827
B profile				
Bilateral	31	12	20	.07
Focal	2	4	2	
A lines	10	13	6	
EF eyeball method				
Severe LV dysfunction	19	0	6	.000
Moderate LV dysfunction	9	2	8	
Normal	13	29	14	
Need for ABG analysis	2	13	16	.000
Dilated RV	15	7	15	.06
Increased kidney echotexture	13	12	15	.144

*ECG* electrocardiogram, *EF* ejection fraction, *LV* left ventricle, *ABG* arterial blood gas, *RV* right ventricle

### Concordance between emergency diagnosis and final diagnosis

The concordance between initial at admission diagnosis and final hospital diagnosis at discharge was analyzed by Cohen’s Kappa test. There is agreement in diagnosis in 88% of patients. The measure of agreement Kappa = 0.805 (*p* = .000). The level of agreement between emergency diagnosis using our diagnostic strategy and final hospital diagnosis is statistically significant.

## Discussion

Our study shows 60 out of 68 patients (88%) received correct disease-specific treatment using the above diagnostic strategy with agreement in ED diagnosis and final discharge diagnosis. The measure of agreement Kappa is 0.805 (*p* .000) where the level of agreement is statistically significant. Patients who expired were excluded for evaluation of diagnostic strategy as many of these expired within 48 h of presentation and were not able to complete their in-hospital evaluation. Most of the discrepancies occurred in patients with acute RV failure if it was due to an acute event like pulmonary embolism or acute exacerbation of chronic pulmonary artery hypertension and in patients having additional component of COPD in ADHF patients. Two studies published in 2014, similar to the present study, assessed the impact of multi-organ POCUS, in addition to history and physical examination, on the accuracy of treating the patient. In a randomized controlled trial (RCT) where patients were randomly assigned to initial assessment with and without point-of-care ultrasonography (POCUS), Pirozzi et al. found that the rate of discordance between initial and final diagnosis was 5% in the POCUS group compared to 50% in the control group [11]. Lauresen et al. found a proportion of correct presumptive diagnosis in the POCUS group of 88% compared to 63.7% in the control group, a significant difference [18].

The most common diagnosis for dyspnea in this study was ADHF (43%). Sensitivity and specificity of the diagnostic strategy used in this study to identify ADHF was 97.3 and 93.3%, respectively. With regard to the test performance characteristics of POCUS as a stand-alone test for ADHF, Kajimoto et al. found a sensitivity and specificity of 94 and 91% [9] and Russell et al. reported sensitivity and specificity of 83 and 83% [9, 10]. As opposed to the more comprehensive and time-consuming echocardiography protocols used by other investigators [19, 20], the echocardiography component of our study protocol simply focused on ejection fraction by gross visual estimation [21, 22] (an adopted method by ACEP for emergency cardiac USG to assess global LV systolic function), presence or absence of pericardial effusion, and right ventricular enlargement while in previous studies, they evaluated diastolic function and Doppler evaluation of the heart. To diagnose diastolic heart failure, we have taken a set of parameters like LVH, LA enlargement, bilateral B-lines on lung USG, and dilated IVC along with suggestive clinical signs. Similarly, the lung examination consisted of assessment of ten zones bilaterally. We have attained similar sensitivity and specificity using this abbreviated protocol. Not only is such an abbreviated protocol feasible during initial resuscitation of the sickest dyspneic patients, but it is likely to be more generalizable to non-expert sonographers across all settings.

To date, there have been just a few studies evaluating a multi-organ POCUS protocol similar to our study–combining abbreviated echocardiography, lung USG, and IVC assessment in the setting of undifferentiated dyspnea. In addition, we included renal USG in the present study to assess kidney size and echotexture. The majority of these studies focused strictly on diagnosis of ADHF [9, 10] while the present study went beyond just ADHF diagnosis. Among the study subjects, dyspnea was attributed to ADHF in 43%, COPD exacerbation in 4%, ARDS in 7%, acute pneumonia in 4%, massive pleural effusion in 3%, acute pulmonary embolism in 7%, and AKI with volume overload and metabolic acidosis in 4%. Patients having both cardiac and non-cardiac cause of dyspnea who accounted for 28% of the study population mostly had a diagnosis of ischemic cardiomyopathy (ICMP) with CKD, COPD/ILD with RV failure, and ADHF with pneumonia. In previous studies by Pirrozi et al. and Laursen et al., acute exacerbation of COPD and acute pneumonia constituted 31.3 and 30% of their study population [11, 18]. In the PRIDE study, COPD and pneumonia constituted 25 and 10.7%, respectively [23]. This difference can be attributed to the fact that most of the COPD and pneumonia patients were OP visits rather than ED visits at our setting and we have not enrolled known COPD patients for whom treating physician lists no other possible diagnosis.

On logistic regression analysis of, at-admission patient characteristics, IVC diameter, EF by eyeball method, and lung sliding showed independent association between cardiac and non-cardiac diagnosis. Among the clinical variables, h/o fever and cough, and jugular venous distension showed independent association. Abnormal ECG and Boston criteria were not independently helpful to differentiate between cardiac and non-cardiac diagnosis. A study by Prosen et al. showed there is significant difference in modified Boston criteria for HF score between cardiac (mean 10.9 ± 1.8) and pulmonary (4.6 ± 1.2) patients [19], while in our study, it showed little difference in scores between cardiac (mean 8.07 ± 1.3) and non-cardiac group (6.86 ± 1.15), questioning the reliability of score in differentiating HF from non-cardiac causes. This difference may be because we included patients with pulmonary, metabolic, and other systemic causes of dyspnea in our non-cardiac group in contrast to other studies which included only pulmonary as non-cardiac.

While it is well established that the presence of AIS is fairly sensitive for detecting ADHF [24], it is possible to have AIS without ADHF. B-pattern alone was not statistically significant between cardiac and non-cardiac groups as interstitial pneumonitis, pulmonary fibrosis, and ARDS will also show a similar pattern. Combined lung-cardiac-IVC USG allowed us to differentiate accurately between these groups. Pleural effusion did not add to B-profile in identifying ADHF in our study as it was in LUCUS protocol study. We faced certain diagnostic challenges in differentiation between diastolic heart failure and pulmonary pathology as both can show B pattern, i.e., to distinguish between wet and dry B-lines. The entire clinical picture helped us in reaching the diagnosis such as - a hypertensive with LVH and LA enlargement with B-pattern was in favor of diastolic heart failure and normotensive with h/o chronic respiratory disease and B-pattern with dirty appearing lungs (fragmented pleural line, subpleural abnormalities) was suggestive of a pulmonary pathology.

We included renal USG in the study as the prevalence of renal failure is high in our setting. Twelve percent of our study population had kidney size of < 9 cm. Combining renal USG to cardiopulmonary USG provided additional diagnostic data in our study population. We did not included BNP in our study as it can be elevated in the setting of CHF when an etiology other than ADHF actually accounts for the acute dyspnea and questionable economic gains and patient benefits of subjecting every patient with dyspnea to BNP assay as diagnostic uncertainty exist with mid-level BNP values [25, 26]. One of the unique features of the present study was that previous studies have included only cardiac and pulmonary causes of dyspnea, but we have also included non-cardiopulmonary causes including renal and metabolic causes fitting more into real-world scenario.

A significant proportion of our patients (28%) presenting with dyspnea had both cardiac and non-cardiac cause. There is a significant increase in morbidity in these patients compared to single cause of dyspnea with increase in time to relief of dyspnea (median 36 h) and hospital LOS (mean 11.5 days). So, it is important to have comprehensive search for all the major causes of dyspnea in every patient as significant number of them could be having multiple disease processes responsible for their symptoms.

Our study has limitations, the first being a small sample size. Patients were enrolled by a single physician trained in ultrasonography. All consecutive patients presenting with dyspnea to ED were not enrolled limited by the availability of enrolling physician. Because of the small sample size, some causes of dyspnea resulted in low recurrence, limiting the reproducibility of data relative to the ability of ultrasound in detecting them. We did not find pneumothorax and cardiac tamponade cases in our case series.

The ED physician sonographer could be influenced by a suggestive clinical presentation as the sonographer is not blinded to patient clinical findings. The primary endpoint was the diagnosis on the patient discharge summary. Although the analysis has been made by two independent physicians, a cardiologist and a pulmonologist, this criterion could be questionable because the final diagnosis was based on a body of evidence including ED diagnosis.

## Conclusions

The present study concludes that integrating focused multiorgan USG by lung-cardiac-IVC and renal ultrasound into routine clinical evaluation for evaluating patients with dyspnea has a higher accuracy for differentiating causes of dyspnea in ED. This strategy can be adopted even in resource-limited setting with training in multiorgan USG, thereby making it widely applicable to patients presenting with dyspnea to ED. More studies are required which should include non-cardiopulmonary causes along with cardiopulmonary causes to come to reasonable decision-making process while evaluating dyspnea, which still remains an enigmatic symptom.

## Abbreviations

ADHF: Acute decompensated heart failure
AIS: Acute interstitial syndrome
BNP: Brain natriuretic peptide
CKD: Chronic kidney disease
COPD: Chronic obstructive pulmonary disease
ED: Emergency department
EF: Ejection fraction
EPSS: E-point septal separation
ILD: Interstitial lung disease
IVC: Inferior vena cava
LV: Left ventricle
LVH: Left ventricle hypertrophy
POCUS: Point-of-care ultrasonography
RA: Right atrium
RV: Right ventricle
SD: Standard deviation
USG: Ultrasonography
